# The Medical Association of Georgia

**Published:** 1882-06

**Authors:** 


### Societies.

### THE MEDICAL ASSOCIATION OF GEORGIA.

*Reported for the Atlanta Medical Rejister.*

The Medical Association of Georgia convened in the senate chamber, Atlanta, April 19th, 1882.

In the absence of the retiring president and both of the vice-presidents, the body was called to order by Dr. James B. Baird, chairman of the committee of arrangements. After prayer by Rev. H. H. Tucker, D.D., and an address of welcome by Dr. James F. Alexander, the President, Dr. W. F. Holt, took the chair and proceeded to deliver the annual address. Among the suggestions contained in this admirable paper, the two most emphasized by the speaker, were the necessity for a state inebriate asylum and for an increase in the annual dues of the members. The Association subsequently acted favorably upon both of these recommendations—a committee was appointed with instructions to memorialize the general assembly on the importance of establishing such an institution, and the amount of dues was fixed at three dollars instead of one and two dollars, as heretofore.

The roll showed that about one hundred and thirty members were in attendance, and from the very moment of assembling the large audience evinced an enthusiastic interest in the proceedings. The following programme was reported by the committee of arrangements and was strictly conformed to during the session :

*First Day—Wednesday—Morning.*—Convene in the senate chamber at eleven o'clock.

*Opening Exercises.*—Prayer by Rev. H. H. Tucker, D.D. Address of welcome by James F. Alexander, M.D., in behalf of the committee of arrangements. President's annual address. Regular order. Adjournment at one o'clock.

*Afternoon.*—Convene at three o'clock. Regular order. Adjournment at the pleasure of the Association.

*Evening.*—Receptions by the Governor at the executive mansion, and by Mr. J. H. Porter at his residence on Peachtree street.

*Second Day — Thursday — Morning.* — Convene at ten o'clock. Regular order. Orator's address at twelve o'clock. Adjournmeut at one o'clock.

*Afternoon.*—Convene at three o'clock. Regular order. Adjournment at the pleasure of the Association.

*Evening.*—Grand banquet at the Markham House.

*Third Day—Friday—Morning.*—Convene at ten o'clock. Regular order. Adjournment at one o'clock.

*Afternoon.*—Convene at three o'clock. Regular order. Adjournment at four o'clock. Excursion to Sweetwater, over the Georgia Pacific Railroad, at half-past four o'clock. Refreshments.

Notes from absentees were read by Dr. Jno. Thad. Johnson, Secretary pro-tem., in the absence of Dr. A. Sibley Campbell, the Recording Secretary, who was detained at home by illness and by a recent bereavement in his family.

The several standing and special committees submitted reports on the various matters intrusted to them.

Among the regular reports, and the great number of voluntary papers, the following were announced by title: "The relative merits of humanized and bovine vaccine virus," by Dr. Eugene Foster. "The therapeutic value of gelseminum," by Dr. Henry F. Campbell. "Catarrhal conjunctivitis," by Dr. Charles W. Hickman. "Hemorrhagic malarial fever," by Dr. R. M. Brown. "Suppurative hepatitis," by Dr. G. G. Roy.

Dr. R. J. Nunn read a paper on "Consumption—its curability and its association with uterine disorders."

"Syphilitic ulceration of the eyelid in the infant," was the title of a paper by Dr. A. W. Calhoun.

"Is typhoid fever contagious ?" was the question discussed in a paper by Dr Philpot.

By Dr. H. F. Scott, a paper entitled "The use of ergot in chronic affections of the throat, lungs and ear, with experiments and reports of cases."

The annual oration, by Dr. J. P. Stevens, was a scholarly address and was listened to with close attention.

Dr. James B. Baird introduced a resolution, which was adopted, with one dissenting voice, re-affirming devotion to the code of ethics of the American Medical Association, and deprecating any attempt to break down or impair this honorable barrier between the regular profession and the vast and varied domain of quackery, and protesting against any departure from the true spirit of the code.

Dr. William O'Daniel read a paper on "Malarial poisoning the cause of hæmaturia."

Dr. Robert Battey, in a most entertaining address, described his visit to London during the session of the International Medical Congress last summer. He exhibited beautiful engravings of many of the attractive places which he visited, and enlivened the recitation by numerous personal reminiscences and by characteristic allusions to many of the great medical men of the old world.

A resolution, by Dr. R. J. Nunn, providing for life membership in the Association was adopted, and the fee was fixed at thirty dollars.

The following officers were elected by the adoption of the report of the committee on nominations: For President, Dr. K. P. Moore; Vice-Presidents, Dr. A. J. Whitehead and Dr. F. R. Calhoun; Treasurer, Dr. E. C. Goodrich; Censor, Dr. A. W. Calhoun. The secretary is elected for a term of five years; Dr. A. Sibley Campbell ably fills that important office.

Dr. H. H. Battey was appointed orator.

Dr. D. O. C. Heery was expelled from the Association on the charge of advertising to treat special diseases.

The more serious work of the session was interspersed by seasons of social relaxation, and while this variety did not interfere with the regular order of business, or with the principal object of the assemblage, it contributed no little, we are constrained to believe, toward the success of the meeting, and to the actual benefit which the members present received. The Association adjourned on the third day, to meet at Athens in April next.

